# Expression of Bioactive Chemerin by Keratinocytes Inhibits Late Stages of Tumor Development in a Chemical Model of Skin Carcinogenesis

**DOI:** 10.3389/fonc.2019.01253

**Published:** 2019-11-15

**Authors:** Ingrid Dubois-Vedrenne, Olivier De Henau, Virginie Robert, Francina Langa, Joaquim Javary, Diana Al Delbany, Olivier Vosters, Edgar Angelats-Canals, Maxime Vernimmen, Souphalone Luangsay, Valérie Wittamer, Marc Parmentier

**Affiliations:** ^1^IRIBHM and Welbio, Université Libre de Bruxelles, Brussels, Belgium; ^2^Centre d'Ingénierie Génétique Murine (CIGM), Institut Pasteur, Paris, France; ^3^Ogeda S.A., Gosselies, Belgium

**Keywords:** chemerin, CMKLR1, ChemR23, chemical carcinogenesis, squamous cell carcinoma

## Abstract

Chemerin is a multifunctional protein acting mainly through the G protein-coupled receptor ChemR23/CMKLR1/Chemerin_1_. Its expression is frequently downregulated in human tumors, including in melanoma and squamous cell carcinoma of the skin and anti-tumoral properties of chemerin were reported in mouse tumor graft models. In the present study, we report the development of spontaneous skin tumors in aged ChemR23-deficient mice. In order to test the potential therapeutic benefit of chemerin analogs, a transgenic model in which bioactive chemerin is over-expressed by basal keratinocytes was generated. These animals are characterized by increased levels of chemerin immunoreactivity and bioactivity in the skin and the circulation. In a chemical carcinogenesis model, papillomas developed later, were less numerous, and their progression to carcinomas was delayed. Temporal control of chemerin expression by doxycycline allowed to attribute its effects to late stages of carcinogenesis. The protective effects of chemerin were partly abrogated by ChemR23 invalidation. These results demonstrate that chemerin is able to delay very significantly tumor progression in a model that recapitulates closely the evolution of solid cancer types in human and suggest that the chemerin-ChemR23 system might constitute an interesting target for therapeutic intervention in the cancer field.

## Introduction

Chemerin, also known as tazarotene-induced gene 2 (*TIG2*) or retinoid acid receptor responder 2 (*RARRES2*), is a secreted protein of 16 kDa (1). It was isolated from human inflammatory fluids as the natural ligand of the previously orphan G protein-coupled receptor ChemR23 (2) also termed Chemokine like receptor 1 (*CMKLR1*) or Chemerin_1_. Liver and adipose tissue are reported as major sites of chemerin production, but the protein is expressed in most tissues, including adrenal gland, placenta, pancreas, lung, and skin (2–5). Chemerin is secreted as an inactive precursor, prochemerin, which is present at nanomolar concentrations in plasma. Prochemerin requires the proteolytic cleavage of 6 or 7 amino acids from its C-terminus for becoming bioactive. This maturation step is mediated by neutrophil-derived serine proteases (elastase and cathepsin G) and proteases of the coagulation and fibrinolytic caiscades (6–8). ChemR23 is coupled to the Gi/o family of G proteins, and chemerin binding to the receptor inhibits cAMP accumulation, induces intracellular calcium mobilization, triggers the mitogen-activated protein kinase (MAPK) cascade and recruits β-arrestins, which promote receptor internalization (2, 3, 9). ChemR23 is expressed by macrophages, immature myeloid and plasmacytoid dendritic cells (DC) and natural killer cells (NK), and chemerin was initially described as a chemotactic factor for these cell populations (2, 10, 11). Expression of ChemR23 was later described as well in adipocytes (4, 12), endothelial cells (13) and muscle cells (14), and chemerin considered as an adipokine regulating lipid and glucose metabolism (4, 15). Two additional receptors, *GPR1* and CC-motif chemokine receptor-like 2 (*CCRL2*) (15, 16), were further reported to bind chemerin with nanomolar affinities. GPR1 (also termed Chemerin_2_) is expressed at high level in brain, but also described in placenta, ovary, testis, skin, adipose tissue and skeletal muscle (17–19). Binding of chemerin to GPR1 leads to β-arrestin2 recruitment and efficient receptor internalization, but signaling through calcium mobilization and ERK1/2 phosphorylation is very weak compared to ChemR23 (20, 21). The role of GPR1 remains therefore unclear so far, but it may belong to the functional family of decoy receptors, which bind and internalize chemoattractant molecules, leading to their degradation (22). CCRL2 is an atypical chemerin receptor expressed by mast cells, activated macrophages and dendritic cells, and endothelial cells (16, 23, 24). It binds chemerin with high affinity, but this interaction does not lead to any known intracellular signaling cascade, nor to receptor internalization. It was therefore hypothesized that CCRL2 acts mainly by regulating the local concentration of chemerin, and promoting its interaction with leukocytes displaying ChemR23 at their surface (16).

Generated by proteolysis as a result of neutrophil activation or tissue injury, and recruiting cells involved in innate immunity (NK cells, macrophages) or the induction of adaptive immunity (dendritic cells), chemerin was first considered as a pro-inflammatory agent. In agreement with this hypothesis, the chemerin-ChemR23 system was shown to take part to the recruitment of leukocytes (25) and the development of lesions in psoriasis (26), autoimmune encephalomyelitis (27), and chronic obstructive pulmonary disease (28). However, in other models, an anti-inflammatory role of chemerin was also highlighted (5, 29, 30). As an adipokine, chemerin was described to regulate adipocyte differentiation and the metabolism of lipids and carbohydrates. Plasma (pro)chemerin levels have been correlated with a number of parameters of the metabolic syndrome, including body mass index, plasma triglycerides, and blood pressure (4, 12, 31).

Changes in chemerin expression have been described in various tumor types (32). Plasma (pro)chemerin levels were increased in patients with gastric cancer (33), colon carcinoma (34), and grade III/IV glioma (35). However, many studies have shown a down-regulation of prochemerin expression in tumors compared to normal tissue, such as in squamous cell carcinoma (SCC) of the skin (36), lung and colon cancer, and melanoma (37). In hepatocellular carcinoma, prochemerin expression was inversely correlated to tumor size, histological grade and aggressiveness (38).

The tumor microenvironment (stromal cells, the extracellular matrix and recruited leukocyte populations) is well-known to influence many aspects of cancer development, including survival and growth of tumor cells, maintenance of the cancer stem cell niche, epithelial to mesenchymal transition, migration of tumor cells, and angiogenesis (39). Intercellular communication between tumor cells and the stroma is driven by a complex network of cytokines, chemokines, growth factors, and matrix remodeling enzymes, in the context of major perturbations of the physical and biochemical properties of the tissue. Considering its role as a chemoattractant factor for leukocyte populations endowed with pro- or anti-tumoral properties (M1 and M2 macrophages, DC, NK cells), chemerin is an obvious candidate player in the regulation of tumoral microenvironment. An anti-tumoral role of chemerin was described in a mouse tumor graft model (B16 melanoma) and attributed to the recruitment of effector NK cells, suggesting that it may act as an endogenous tumor suppressor (37).

In the present study, we tested the potential therapeutic effect of chemerin in a mouse model of tumor progression mimicking the natural development of solid cancers in human. We generated for this purpose a transgenic model expressing a bioactive form of chemerin in basal keratinocytes and investigated the consequences of this expression in a chemical model of skin carcinogenesis. In these mice, papillomas developed later, their number was lower and they progressed much more slowly to carcinomas. This effect was restricted to the late steps of the carcinogenic process and reversed by ChemR23 invalidation.

## Materials and Methods

### Mice

C57BL/6J mice (RRID:IMSR_JAX:000664) were purchased from Janvier. The ChemR23 knockout (ChemR23^−/−^) mouse line (RRID:MGI:4430513) was initially obtained from Deltagen and backcrossed into the C57BL/6J background for over 20 generations. Heterozygotes (ChemR23^+/−^) were intercrossed to generate F2 ChemR23^−/−^ mice and their wild-type (WT) controls. The mouse line expressing the tetracycline transactivator under control of the K5 promoter (K5-tTA) was described previously (40). Mouse lines expressing the most bioactive form of mouse chemerin (1-157 of isoform 1, ending with -FAFS at the C-terminus) under control of tetracycline response elements (TRE) were generated locally on the C57BL/6J background, and selected for chemerin expression after breeding with K5-tTA mice (K5-chemerin model). Mice were maintained in a specific pathogen-free environment with environmental enrichment and unlimited access to food and water and, except otherwise stated, were used between 6 and 10 weeks of age. In all experiments, animals were age-matched and distributed randomly into groups.

All animal experiments were conducted in accordance with European guidelines and local regulations, and approved by the local ethics committee (Commission d'Ethique du Bien-Etre Animal, CEBEA) of the ULB Medical School. All efforts were made to minimize suffering.

### Western Blotting

Tissues were lysed by incubation for 5 min at 94°C in 62.5 mM Tris-HCl pH 6.8 containing 3% SDS, and insoluble material removed by centrifugation at 15,000 g for 5 min. The protein concentration was measured by using the bicinchoninic acid (BCA) assay kit (Pierce Thermo Scientific) and the extract adjusted to 62.5 mM Tris-HCl pH 6.8, 5% β-mercaptoethanol, 3% SDS, 10% glycerol and 0.01% bromophenol blue. Protein lysates (10 μg proteins per sample) were separated on 15% SDS-PAGE gels and transferred onto nitrocellulose membranes. The membranes were incubated in blocking buffer (PBS containing 5% BSA and 0.1% Tween 20) for 1 h at room temperature, then probed overnight at 4°C with goat anti-chemerin (R&D Systems, AF2325, RRID:AB_2175558, 1/250) and rabbit anti-GFP (Molecular Probes, A6455, RRID:AB_221570, 1/2000) antibodies diluted in PBS containing 3% BSA and 0.1% Tween 20. Membranes were washed three times in PBS containing 0.1% Tween 20, then probed with HRP-conjugated anti-rabbit or anti-goat antibodies (Thermo Fisher Scientific, 31460, RRID:AB_228341 and 31402, RRID:AB_228395, 1:50,000 in the same buffer) for 1 h at room temperature. Proteins were visualized by enhanced chemiluminescence using the SuperSignal West Pico HRP substrate (Pierce Thermo Scientific).

### Chemical Carcinogenesis Model

The DMBA/TPA chemical carcinogenesis model was performed as previously described (41). Briefly, 8-week-old mice (all of C57BL/6J background) were treated under anesthesia (4% isoflurane) during the first and the seventh week with 9,10-dimethyl-1,2-benzanthracene (DMBA, Sigma, 50 μg in 200 μl acetone, applied on shaved skin three times at 2 days' interval), and with 12-O-tetradecanoyl phorbol-13-acetate (TPA, Sigma, 4 μg in 200 μl acetone, applied twice a week) from weeks 2 to 6 and from week 8 onwards. The number of tumors was recorded every other week, and their size measured with a caliper. Animals were sacrificed whenever a tumor reached a diameter of 10 mm. When necessary, the expression of chemerin in K5-chemerin mice was switched off by administration of doxycycline (Sigma, D9891, 2 mg/mL) in drinking water.

### Flow Cytometry Analyses

Skin samples were cut into small fragments (about 1 mm^3^) and digested in HBSS medium containing 5% fetal bovine serum and 3.5 mg/mL collagenase for 1 h 30 at 37°C on a rocking plate. Collagenase D activity was blocked by the addition of 5 mM EDTA, the cell suspension was rinsed with PBS and tissue debris removed by filtering through a 70-μm nylon mesh. Lymph nodes and thymus were crushed in RPMI medium and the cell suspension filtered through a 70-μm nylon mesh. Spleen was treated similarly but red blood cells were lysed with ammonium-chloride-potassium (ACK). Single-cell suspensions were incubated for 30 min at 4°C with anti-CD16/CD32 Fc Block (eBioscience, 14-0161-86, RRID:AB_467135) and a mixture of antibodies in FACS buffer (PBS containing 1% FCS, 1 mM EDTA and 0.1% NaN_3_). Flow cytometry analysis was performed on a LSRFortessa instrument (BD Biosciences) and analyzed using the FlowJo software. The antibodies used were directed to CD45 (47-0451, RRID:AB_1548781 and 17-0451-83, RRID:AB_469393), NK1.1 (12-5941-82, RRID:AB_466050), CD3 (17-0032-82, RRID:AB_10597589) and CD4 (48-0041, RRID:AB_10718983) from eBioscience, and CD8 (551162, RRID:AB_394081), CD11b (550993, RRID:AB_394002 and 553311, RRID:AB_394775), CD11c (550261, RRID:AB_398460), B220 (553088, RRID:AB_394618 and 552772, RRID:AB_394458) and Ly6G (551461, RRID:AB_394208 and 551460, RRID:AB_394207) from BD Pharmingen.

### Histological Procedures

Back skin was pre-fixed for 2 h in 4% paraformaldehyde, embedded in OCT (Tissue Tek, Sakura) and sectioned at 8 μm using a Leica cryostat. Whole newborn mice were pre-fixed in 4% paraformaldehyde for 48 h, incubated in 30% sucrose for 48 h and embedded in OCT before performing 12 μm-thick sagittal sections. Tumors were embedded in OCT and sections post-fixed in acetone for 10 min at room temperature. For immunofluorescence staining, the sections were blocked with 5% horse serum, incubated for 2 h 30 min at room temperature with rat anti-CD45 (BD Biosciences, 553077, RRID:AB_394607, 1/250), rat anti-β4 integrin (BD Biosciences, 553745, RRID:AB_395027, 1/200), rabbit anti-keratin 5 (Covance, Cat# PRB-160P-100, RRID:AB_291581, 1/4000) or rabbit anti-keratin 10 (Covance, PRB-159P-100, RRID:AB_291580, 1/1000) antibodies, and then for 2 h at room temperature with a rhodamine red-X-conjugated anti-rat IgG (Jackson ImmunoResearch, 712-295-150, RRID:AB_2340675, 1/400) or Alexa Fluor 488 conjugated anti-rabbit IgG (Thermo Fisher Scientific, A-21206, RRID:AB_2535792, 1/400) secondary antibodies. Nuclei were stained with Hoechst 33342 (Molecular Probes, H3570, 1:4000) and slides were mounted in DAKO mounting medium supplemented with 2.5% 4-diazabicyclo[2.2.2]octane (DABCO, Sigma). For EGFP immunohistochemistry, sections were blocked with 5% horse serum, incubated overnight at 4°C with a chicken anti-GFP antibody (Abcam, ab13970, RRID:AB_300798, 1/4000), for 1 h at room temperature with a HRP-conjugated anti-chicken secondary antibody (Thermo Fisher Scientific, 31401, RRID:AB_228385, 1/2500) and stained with 3,3′-diaminobenzidine (DAB, ImmPACT, Vertorlabs) as a peroxidase substrate. Slides were counter-stained by hematoxylin-eosin and mounted in Entellan (Merck). Images were acquired using a Zeiss LSM 780 confocal microscope or a Zeiss AxioImager Z1 (Carl Zeiss). They were analyzed and their contrast adjusted with the ImageJ software.

For chemerin immunodetection, skin samples were collected and fixed with 4% PAF in PBS pH 7.4 for 2 h before paraffin embedding. Five micrometers sections were rehydrated and washed in PBS. An enzymatic antigen retrieval was performed by incubating the slides in 50 mM Tris-HCl pH 8.0 containing 1 mM EDTA and 20 μg/ml proteinase K for 5 min before rinsing the slides in PBS. Afterwards, the slides were blocked with PBS containing 1% BSA, 0.5% normal goat serum and 0.05% Triton-X100 for 1 h at room temperature before an overnight incubation at 4° with a rabbit polyclonal anti-chemerin antibody (Bioss, #bs-1501R, dilution 1/50 in blocking buffer). A 1 h incubation at room temperature with a Cy5-conjugated goat anti-rabbit secondary antibody (Thermo Scientific, #A10523, dilution 1/200 in Blocking buffer) and labeling of the nuclei with Hoechst (1/2000 in Blocking Buffer) was performed before mounting the slides with FluorSave Reagent (Calbiochem; #345789). Images were collected with a Leica DMI6000 microscope.

### Immunoassays

The blood of K5-chemerin and control mice was collected and centrifuged at 300 g for 10 min. We assessed the plasma chemerin concentration by using a mouse chemerin DuoSet ELISA kit (R&D Systems, DY2325), the plasma C-Reactive Protein (CRP) by using a mouse C-Reactive Protein/CRP Quantikine ELISA kit (R&D Systems, MCRP00), the plasma TNF-α concentration by using a mouse TNF-α DuoSet ELISA kit (R&D Systems, DY410) and the plasma IL-6 concentration by using a mouse IL-6 DuoSet ELISA kit (R&D Systems, DY406), following the manufacturer's instructions.

### Chemerin Bioactivity Assay

Blood from four WT or K5-chemerin mice was collected, pooled and centrifuged at 300 g for 10 min and the plasma was loaded onto a 1 mL HiTrap Heparin HP column (GE Healthcare) eluted by a NaCl gradient (20–700 mM, 10 mM/min) in a 20 mM Na phosphate buffer pH 7.0. All solutions contained a cocktail of protease inhibitors (cOmplete with EDTA, Roche). Fractions were collected and the chemerin bioactivity was determined by assaying the functional response of mouse ChemR23 in an aequorin-based calcium mobilization assay (6). Briefly, CHO-K1 cells (ATCC, CCL-61, RRID:CVCL_0214) co-expressing mouse ChemR23, apoaequorin, and Gα16 (or control CHO-K1 cells co-expressing apoaequorin and Gα16), were incubated for 3 h in the dark in DMEM containing 5 μM coelenterazine H (Thermo Fisher Scientific). 5.10^4^ cells in a volume of 50 μl were added to wells containing different concentrations of recombinant mouse chemerin, or blood-derived heparin column fractions with unknown chemerin concentrations, and luminescence was recorded for 20 s in a Packard luminometer. Results (as luminescence units) were normalized to the response to 20 μM ATP, and the parameters of the dose-response curves were determined with the Prism software using non-linear regression applied to a sigmoidal dose-response model. This assay does not take into account the recovery rate of bioactive chemerin during the purification procedure and constitutes therefore a lower estimate of the actual concentration in blood.

### Statistics

Statistical analyses and data graphing were performed using Prism 6 (GraphPad Software). Statistical significance was calculated by Student's *t*-test, Mann-Whitney test, one-way ANOVA, two-way ANOVA or Log-rank test, as indicated. *P* < 0.05 were considered significant.

## Results

### ChemR23 Knockout Mice Develop Spontaneous Skin Tumors

**Figure 1:**
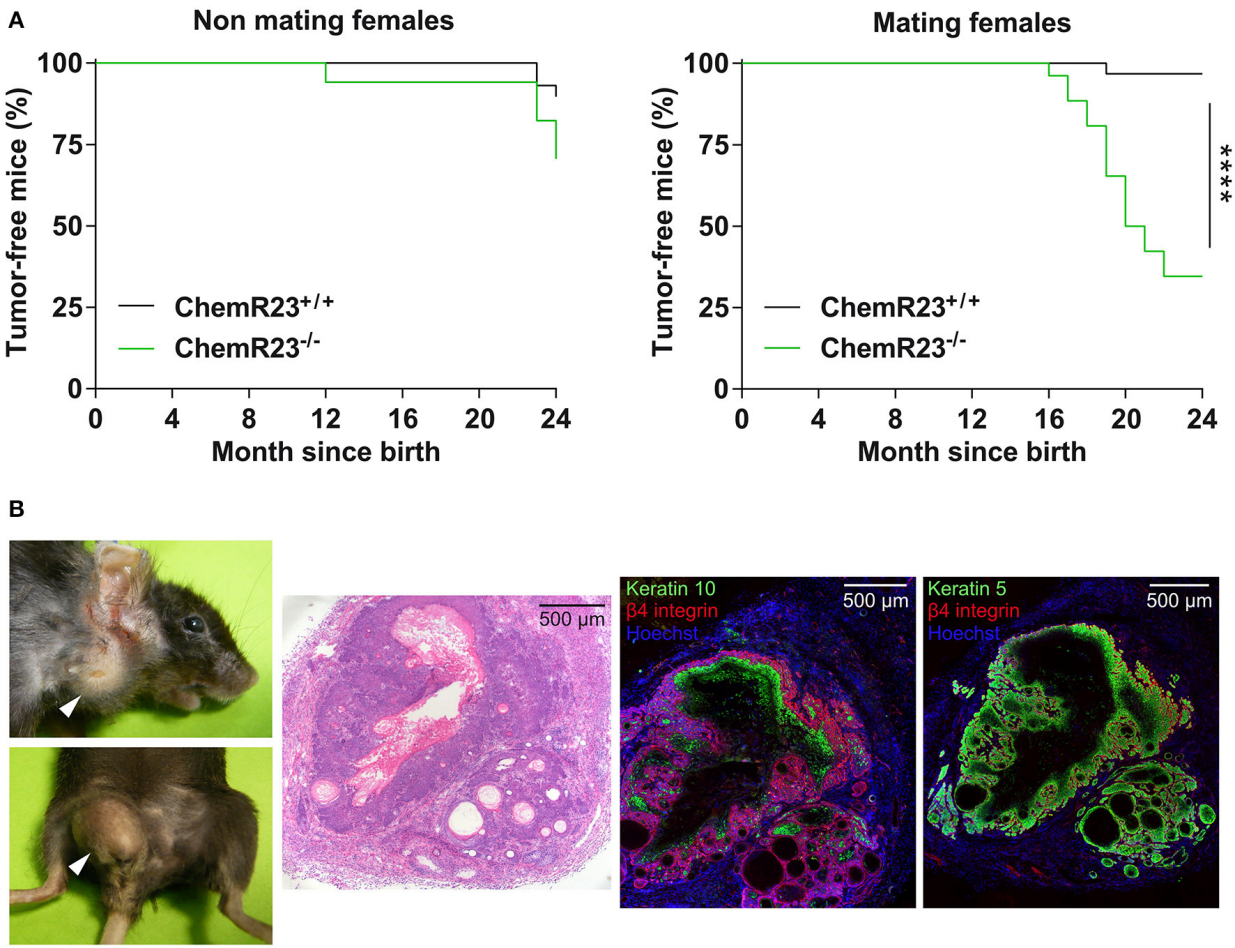
Mice invalidated for ChemR23 develop spontaneous skin tumors. **(A)** Kaplan-Meyer plots representing the percentage of tumor-free mice in cohorts of control and ChemR23^−/−^ female mice kept chronically (right panel, *n* = 30 and 25 for respectively, controls and ChemR23^−/−^) or not (left panel, *n* = 26 and 17 for respectively controls and ChemR23^−/−^) in conditions of mating. The data are analyzed by Log-rank test. ****P < 0.0001. **(B)** Spontaneous skin tumors located in the neck and perineal regions of aged ChemR23^−/−^ female mice (left panels). White arrowheads indicate the location of tumors. Hematoxylin-eosin staining and immunostaining of keratin 10, keratin 5, and β4 integrin in a spontaneous skin tumor (right panels).

A mouse line invalidated for ChemR23 (*Cmklr1*) was used previously to investigate the role of chemerin and its receptor in the mounting of anti-viral responses and as an anti-inflammatory system in lung diseases (29). We observed over time that old mice from these colonies developed with high frequency spontaneous skin tumors (Figures 1A,B). These tumors appeared preferentially on animals prone to chronic skin injury by biting or scratching, such as females maintained in breeding programs for long periods. In this group, the prevalence of tumors reached over 50%, and they developed most often in the neck and perineal regions. Male siblings kept together also developed such tumors, while they were rare in non-mating females (Figure 1A) or male mice kept in individual cages. These tumors were classified as well-differentiated spinocellular carcinomas on a histological basis (42). They contained typical horn pearls, rarely displayed surface ulcerations and the tumor cells expressed keratin 5 (*Krt5*) and β4-integrin (*Itgb4*) in basal layers, and keratin 10 (*Krt10*) in upper layers when present (Figure 1B). No viral inclusions were observed. Such tumors were never encountered in wild-type animals kept in the same environment and housing conditions. These observations suggested that the chemerin/ChemR23 system plays a protective role against the development of skin tumors in the context of chronic lesions and tissue repair processes. Anti-tumoral properties of chemerin were also suggested in the literature, several studies reporting decreased expression of chemerin in human tumors (36, 37). We therefore initiated the testing of this hypothesis in an experimental model of tumor progression.

### Generation of a Mouse Model Expressing Bioactive Chemerin in Basal Keratinocytes

**Figure 2:**
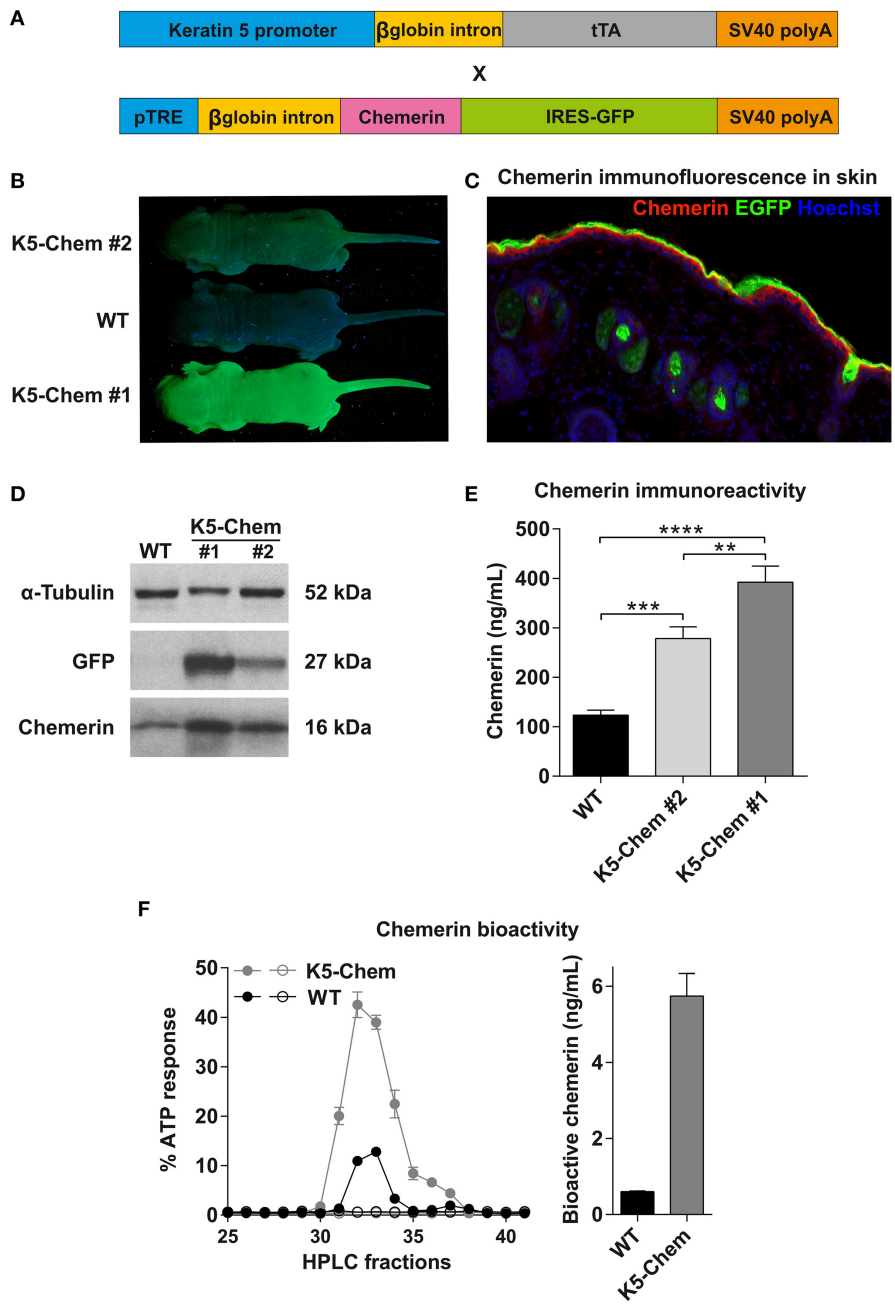
Development of a mouse line expressing bioactive chemerin in keratinocytes. **(A)** Schematic representation of the constructs used to generate the K5-chemerin transgenic mice overexpressing bioactive chemerin and EGFP in keratinocytes. **(B)** Detection of EGFP fluorescence in the skin of newborn mice from two K5-chemerin lines and a control mouse (WT). **(C)** Detection by immunofluorescence of chemerin in a skin section of a K5-chemerin mouse (line ^#^1). **(D)** Detection by Western blotting of chemerin (16 kDa), EGFP (27 kDa) and α-tubulin (52 kDa, as control) in the skin from K5-chemerin (lines ^#^1 and ^#^2) and WT mice. Data are representative of four experiments. **(E)** Measurement by ELISA of chemerin immunoreactivity in plasma from control and K5-chemerin mice from two lines (mean ± SEM, *n* ≥ 5 mice per group). The data are representative of three experiments and analyzed by one-way ANOVA. **P < 0.01; ***P < 0.001, and ****P < 0.0001. **(F)** Measurement of chemerin bioactivity in the plasma of control and K5-chemerin mice (line ^#^1). Pooled plasma was loaded on a heparin column and fractions tested in an aequorin-based intracellular Ca^2+^ mobilization assay, using CHO-K1 cells expressing mouse ChemR23 (closed symbols) or not (open symbols). The activity is expressed as the percentage of the response obtained for 10 μM ATP (left panel, representative experiment out of two). Bar graph representing the total bioactive chemerin level in plasma from control and K5-chemerin mice (right panel). For each condition, two pools of plasma were analyzed (mean ± SEM).

**Figure 3:**
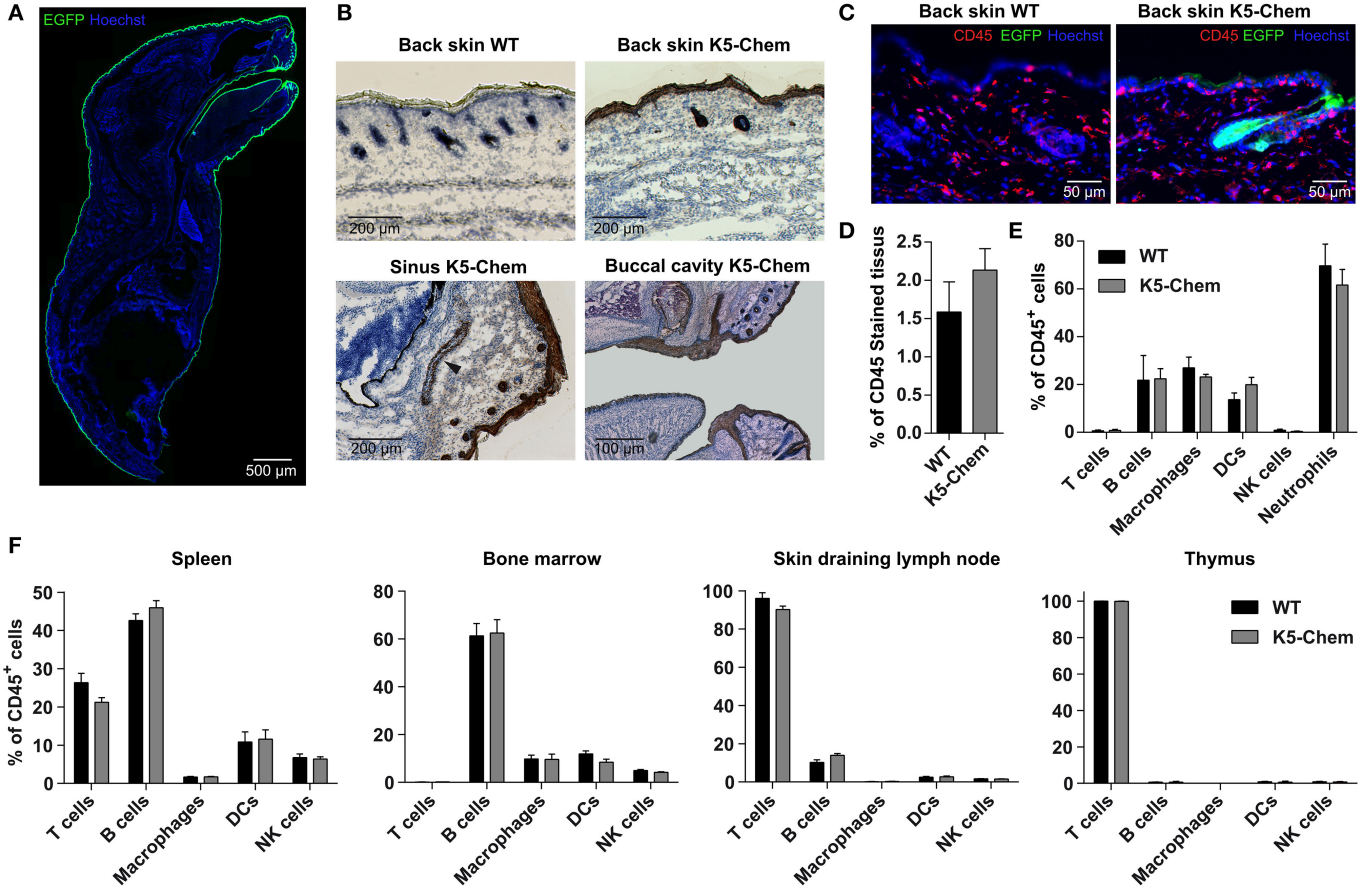
Overexpression of bioactive chemerin in skin does not affect leukocyte populations in skin and lymphoid organs. **(A,B)** Whole newborn mice were pre-fixed for 48 h in 4% paraformaldehyde, embedded in OCT and sagittal sections were made. **(A)** Reconstitution of a sagittal section of a K5-chemerin mouse, with EGFP intrinsic fluorescence in green and cell nuclei stained in blue (Hoechst). **(B)** Cryosections were stained by immunohistochemistry for EGFP (brown) and counter-stained by hematoxylin-eosin. Upper panels show the back skin of control (left) and K5-chemerin (right) mice. Lower panels illustrate sections through sinus cavities (left) and a partial reconstitution of the buccal cavity (right) of K5-chemerin mice. Black arrowhead indicates the location of the sinus. **(C)** Skin cryosections from control (left) and K5-chemerin (right) mice showing CD45 staining (red), EGFP fluorescence (green), and nuclei (Hoechst, blue). **(D)** The relative surface of CD45^+^ staining in skin sections was measured and graphed (mean ± SEM, *n* = 5 mice per group). **(E,F)** Skin samples **(E)**, thymus, skin draining lymph nodes, bone marrow and spleen **(F)** were collected from control and K5-chemerin mice and digested. The cell suspensions were stained with combinations of antibodies and analyzed by flow cytometry. The percentage of CD45^+^ cells and the proportion of various leukocyte subsets (% of CD45^+^ cells) are represented, including T cells (CD3^+^ CD4^+^ and CD3^+^ CD8^+^), B cells (CD3^−^ B220^+^), neutrophils (Ly6G^+^ CD11b^+^) macrophages (CD11b^+^ CD11c^−^), DCs (CD11c^+^) and NK cells (CD3^−^ NK1.1^+^). The results are from a representative experiment (mean ± SEM, *n* = 5 mice per group).

We generated a mouse model, based on the tet-off system, in which a C-terminally truncated bioactive form of chemerin (*Rarres2*) was expressed under control of the keratin K5 promoter. A mouse line expressing the tetracycline transactivator under control of the K5 promoter (K5-tTA) was described previously (40). Keratin K5 is expressed, together with K14 (*Krt14*), in the basal cell layer of the epidermis and of Malpighian epithelia of internal cavities. A bicistronic construct was designed, in which bioactive mouse chemerin (1–157) and EGFP (as a reporter) open reading frames, separated by an IRES sequence, were placed under control of tetracycline response elements (TRE) (Figure 2A). This construct was microinjected into the pronucleus of C57BL/6 fertilized single cell embryos, and the resulting transgenic lines were tested for chemerin and EGFP expression following breeding with K5-tTA mice. Four lines expressed the construct at different levels, and we selected two lines, K5-chemerin #1 displaying strong expression of the transgene, and K5-chemerin #2 characterized by a milder expression level (Figures 2B,D). Chemerin was detected by immunofluorescence in the basal layers of the skin of K5-chemerin mice (Figure 2C). The labeling did not overlap strictly with GFP, as chemerin is secreted while EGFP remains in the cell and concentrates within the upper layers of the epithelium. As expected from the bicistronic structure of the construct, the expression levels of EGFP and chemerin, as determined by Western blotting, were correlated (Figure 2D). The concentration of chemerin in plasma, assayed by ELISA, was significantly increased in the transgenic mouse lines (Figure 2E). Compared to 123 ± 10 ng/mL (7.93 ± 0.64 nM) in WT mice, it reached 278 ± 23 ng/mL (17.9 ± 1.5 nM) in the K5-chemerin #2 line, and 392 ± 32 ng/mL (25.3 ± 2.1 nM) in the K5-chemerin #1 line (Figure 2E). As the chemerin ELISA does not allow to distinguish prochemerin, active chemerin forms, and inactive degradation products, we also assayed chemerin bioactivity in plasma by using an aequorin-based calcium mobilization assay, following concentration and fractionation by a heparin HPLC column (Figure 2F). The concentration of bioactive chemerin was estimated to 0.60 ± 0.01 ng/mL (0.038 ± 0.001 nM) in pooled plasma from WT mice and 5.74 ± 0.59 ng/mL (0.37 ± 0.03 nM) in that of K5-chemerin mice (line #1) (Figure 2F). As part of the activity was certainly lost during the purification procedure, these numbers constitute lower estimates of the actual concentrations of bioactive chemerin in plasma. Nevertheless, these data show that the production of bioactive chemerin by basal keratinocytes results in a significant increase of chemerin immunoreactivity in plasma, but that only a fraction (of at least 2%) retains its bioactivity, as the likely result of C-terminal proteolytic processing. Considering the stronger expression of chemerin in line #1, we used this line in subsequent experiments. The expression of the TRE-chemerin/EGFP transgene was investigated macroscopically on whole organs and microscopically on sections from various tissues, through the fluorescence of EGFP. Expression was found in the epidermis and hair follicles but also in the epithelium of the buccal cavity, esophagus, pharynx and sinuses (Figures 3A,B), all tissues known to express keratin K5 in basal layers.

The primary role attributed to chemerin is that of a chemoattractant factor for leukocyte populations, and the presence of significant concentrations of chemerin bioactivity in organs and blood might be expected to affect the trafficking of these populations. We therefore investigated the distribution of the main leukocyte populations in blood and lymphoid organs, as well as skin, as the main chemerin-producing tissue in the transgenic mice. The blood cell counts were not modified (data not shown). Cryosections of K5-chemerin and control mice were stained with an anti-CD45 antibody, and the percentage of CD45^+^ cells was found similar in both genotypes (Figures 3C,D). Bone marrow, spleen, thymus, inguinal lymph nodes and skin from K5-chemerin and wild type mice were collected and digested, and flow cytometry analysis was performed on the cell suspensions. No significant differences were observed in the numbers or relative proportions of the main immune cell subsets, namely T cells (CD3^+^ CD4^+^ and CD3^+^ CD8^+^), B cells (CD3^−^ B220^+^), macrophages (CD11b^+^ CD11c^−^), NK cells (CD3^−^ NK1.1^+^) and dendritic cells (CD11c^+^) between K5-chemerin and control mice (Figures 3E,F). As chemerin is described as a pro-inflammatory factor, we assayed a few markers of inflammatory status in the blood of K5-chemerin mice. C-reactive protein (CRP) was found unchanged between wild-type (4.4 ± 0.34 μg/mL) and K5-chemerin (4.3 ± 0.30 μg/mL) mice (mean ± SEM, *n* = 5 per group, *p* = 0.94, Student's *t*-test). TNF-α and IL-6 were below the detection threshold (<15 pg/mL) in all samples of both groups. These data suggest that the overexpression of chemerin does not affect significantly the trafficking and distribution of major leukocyte populations, nor the inflammatory status of the mice, at least in SPF conditions. It remains however possible that minor cell populations that were not assayed specifically could be modified in K5-chemerin mice.

### Chemerin Delays the Development of Tumors in a Chemical Carcinogenesis Model

We tested the consequences of chemerin overexpression in skin in a model of chemical carcinogenesis, which recapitulates the various steps of the oncogenic process. The DMBA/TPA model is a classical model of tumor development through the successive stages of initiation, promotion and progression (41, 43, 44). In this model, the mutagen 9,10-dimethyl-1,2-benzanthracene (DMBA) is used to initiate the tumoral process, while repeated skin painting by 12-O-tetradecanoyl phorbol-13-acetate (TPA) stimulates epidermal cell proliferation and a chronic inflammatory status supporting tumor progression. Since C57BL/6 mice are notoriously resistant to this tumor model (41), we added a second DMBA treatment at week seven of the procedure, speeding up the carcinogenesis process, and the development and size of benign (papillomas) and malignant tumors (squamous cell carcinomas) was monitored over time.

**Figure 4:**
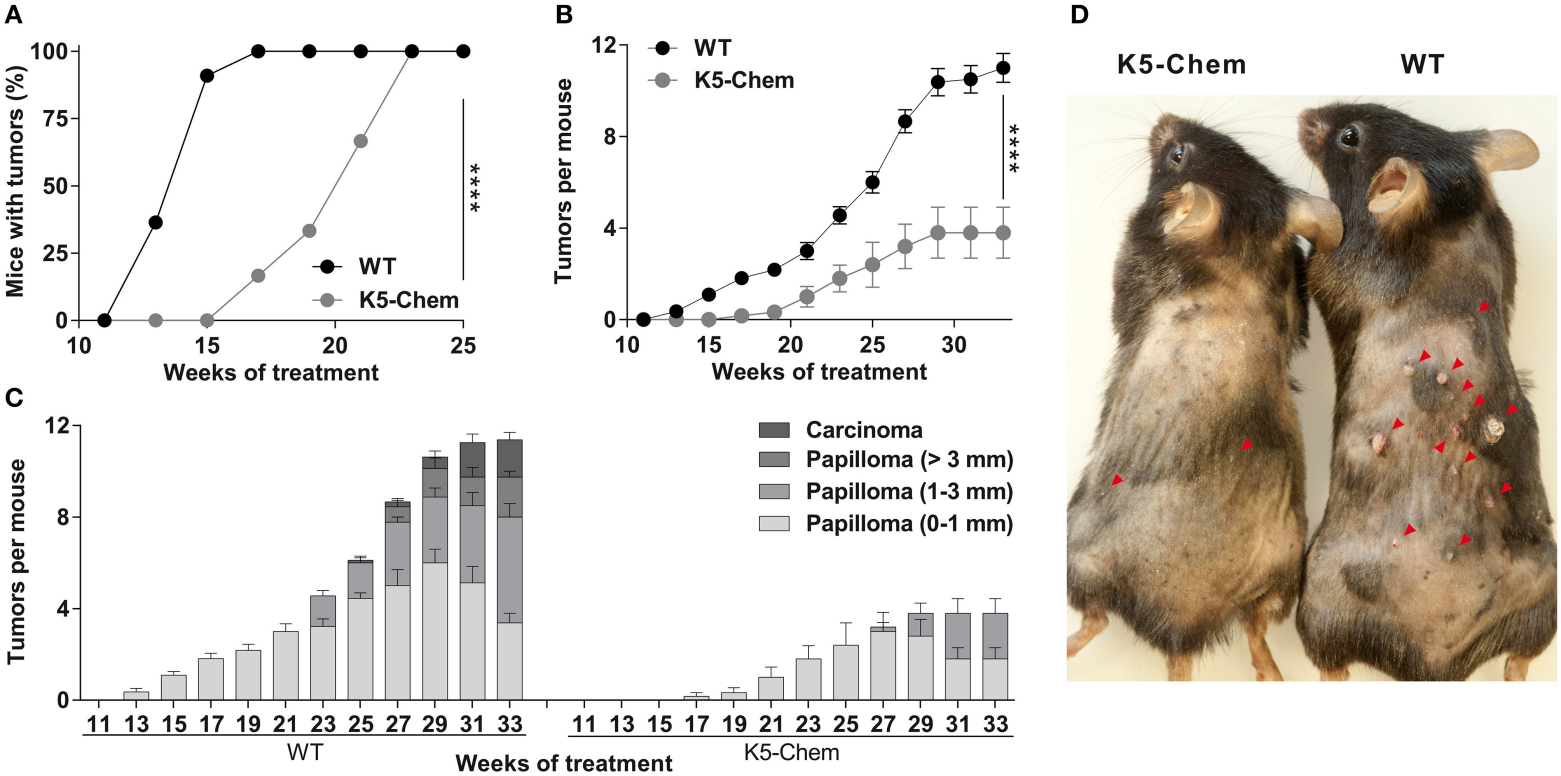
Overexpression of bioactive chemerin in the skin delays tumor progression in a chemical carcinogenesis model. WT and K5-chemerin mice were subjected to the DMBA/TPA chemical carcinogenesis model. **(A)** Percentage of mice with tumors. The data are analyzed by Log-rank test. ****P < 0.0001. **(B,C)** Number of tumors per mouse (**B**, mean ± SEM) and proportion of tumors according to size and stage was recorded every other week (**C**, mean ± SEM). The data are analyzed by Mann-Withney test. The difference in tumor number between wild-type and K5-chemerin mice became significant from week 15 (*P* < 0.001 and *P* < 0.0001 at week 33). **(D)** Representative WT and K5-chemerin mice at week 30 of the DMBA/TPA protocol. Red arrowheads indicate the location of papillomas. The displayed data result from the compilation of three independent experiments with five mice per group in each experiment.

**Figure 5:**
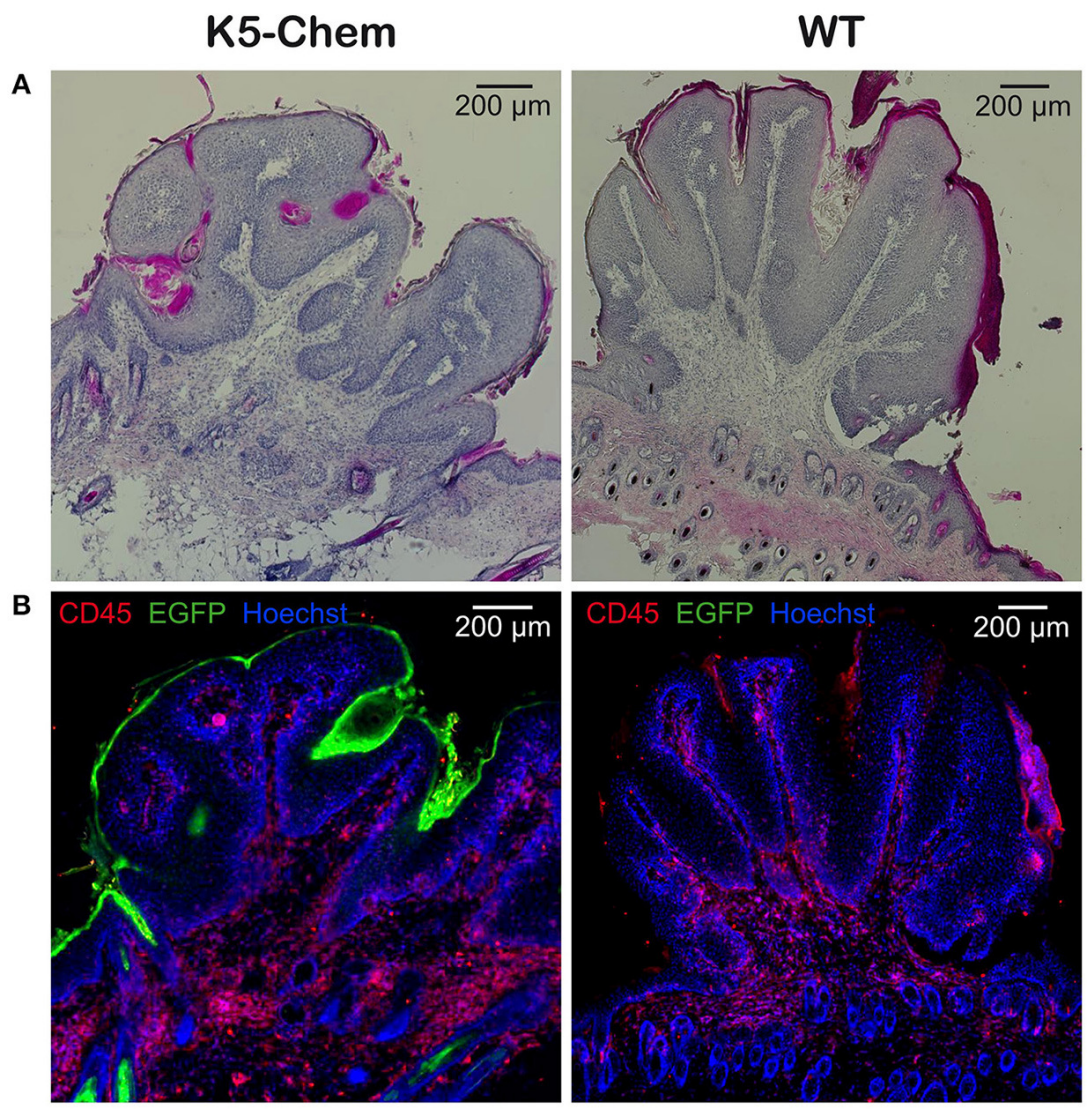
Histology of papillomas from control and K5-chemerin mice under the DMBA/TPA protocol. Reconstitution of papilloma sections collected at week 30 of the DMBA/TPA protocol from K5-chemerin mice (left panels) and WT (right panels). Cryosections were stained with hematoxylin-eosin **(A)** or for CD45 (red), EGFP (green), and nuclei (Hoechst, blue) **(B)**.

Under this protocol, the first papillomas appeared around week 12–13 in WT mice, but not before week 16–17 in K5-chemerin mice (Figure 4A). All mice developed at least one papilloma by week 17 in the WT group and week 23 in the K5-chemerin group. The average number of tumors per mouse remained much smaller in K5-chemerin mice compared to controls (Figure 4B). The progression of tumors was also delayed considerably and few (if any) large papillomas or carcinomas developed in chemerin-expressing mice (Figures 4C,D). The absence of progression to carcinomas is not the consequence of the lower number of tumors overall. In wild-type mice, 28% of the papillomas progressed to large tumors (>3 mm) or carcinomas by the end of the protocol, and the absence of such tumors in the K5-chemerin group is highly significant (*p* < 0.001, Pearson's chi-square test). The histology of the papillomas was similar in both groups, as illustrated in Figure 5. These data demonstrate that chemerin delays the appearance of tumors and the progression of these tumors to malignancy in a model mimicking the natural evolution of many human cancer types in which chronic inflammation plays a major role.

### Chemerin Affects Exclusively the Late Stages of Tumor Progression

**Figure 6:**
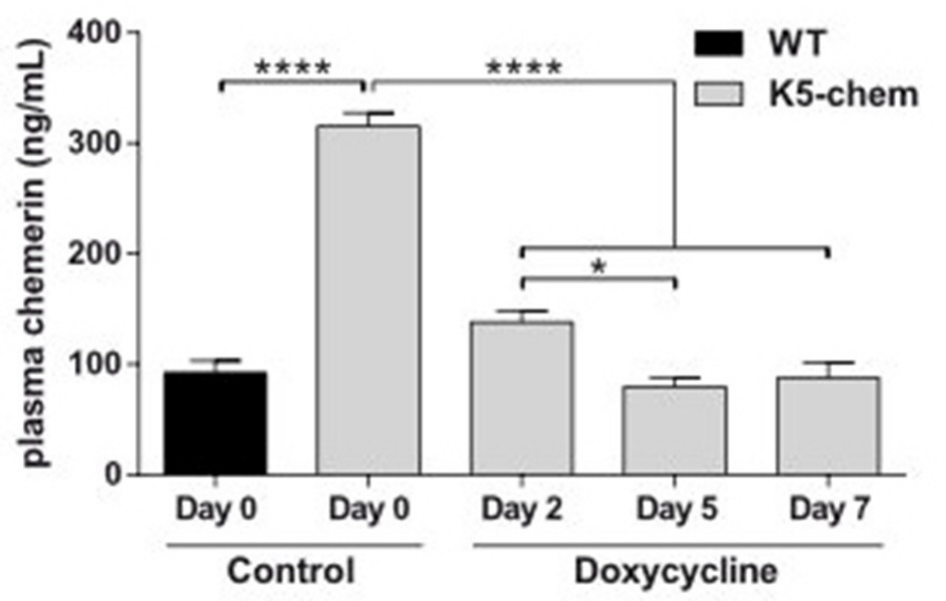
Downregulation of the chemerin-eGFP transgene expression under doxycycline treatment. WT and K5-chemerin mice were treated with doxycycline and the chemerin immunoreactivity was measured by ELISA in plasma at 2, 5, and 7 days of treatment. The results are from a representative experiment out of two (mean ± SEM, *n* = 5 mice per group) and analyzed by one-way ANOVA. *P < 0.05 and ****P < 0.0001.

**Figure 7:**
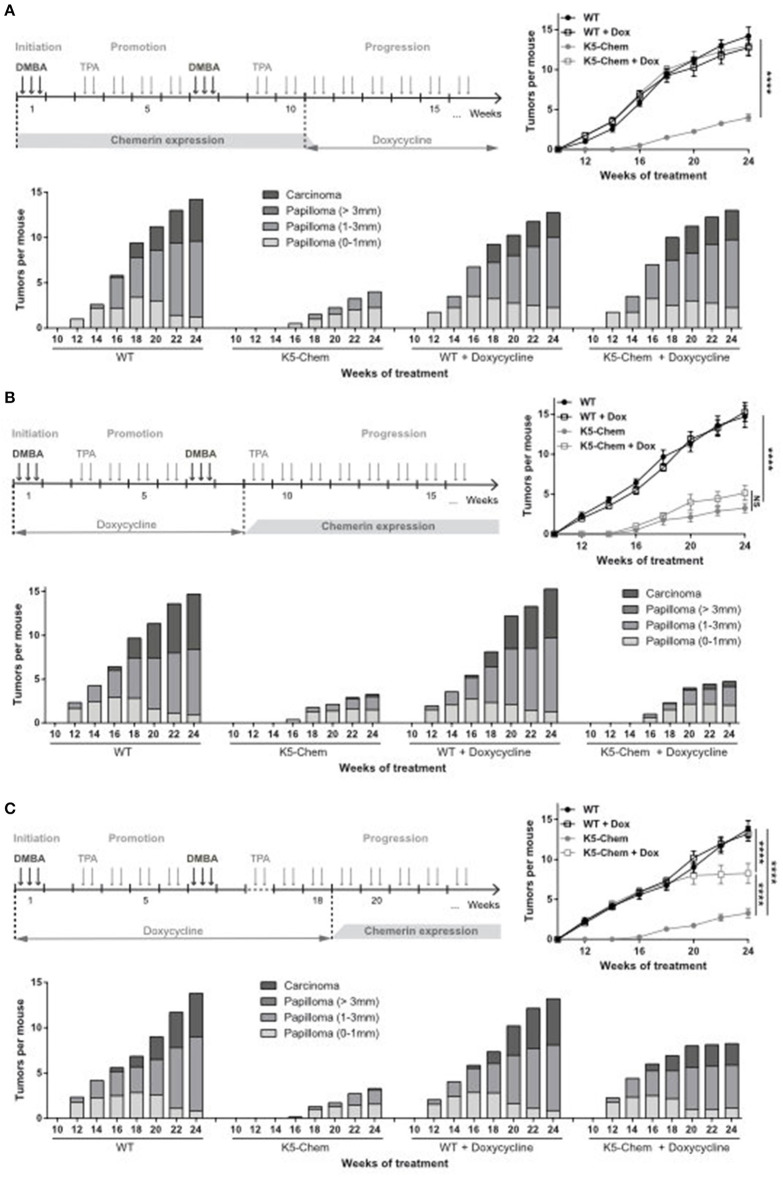
Chemerin expression affects only the late stages of tumor progression. WT and K5-chemerin mice were subjected to the DMBA/TPA chemical carcinogenesis model. They were treated or not during part of the protocol with doxycycline, in order to inhibit expression of the chemerin/eGFP transgene in K5-chemerin mice. Three schemes of doxycycline treatment were used: **(A)** From week 11 up to the end of the protocol, **(B)** from a week before initiation of the protocol to the end of week 8, or **(C)** for a week before initiation of the protocol to the end of week 18. For each treatment scheme, the number of tumors per mouse (right graph, mean ± SEM) and the proportion of tumors according to size and stage (histogram) were recorded every other week. The displayed data result from the compilation of three **(A)** or two **(B,C)** independent experiments with *n* ≥ 6 mice per group in each experiment. The data are analyzed by two-way ANOVA. ****P < 0.0001.

**Figure 8:**
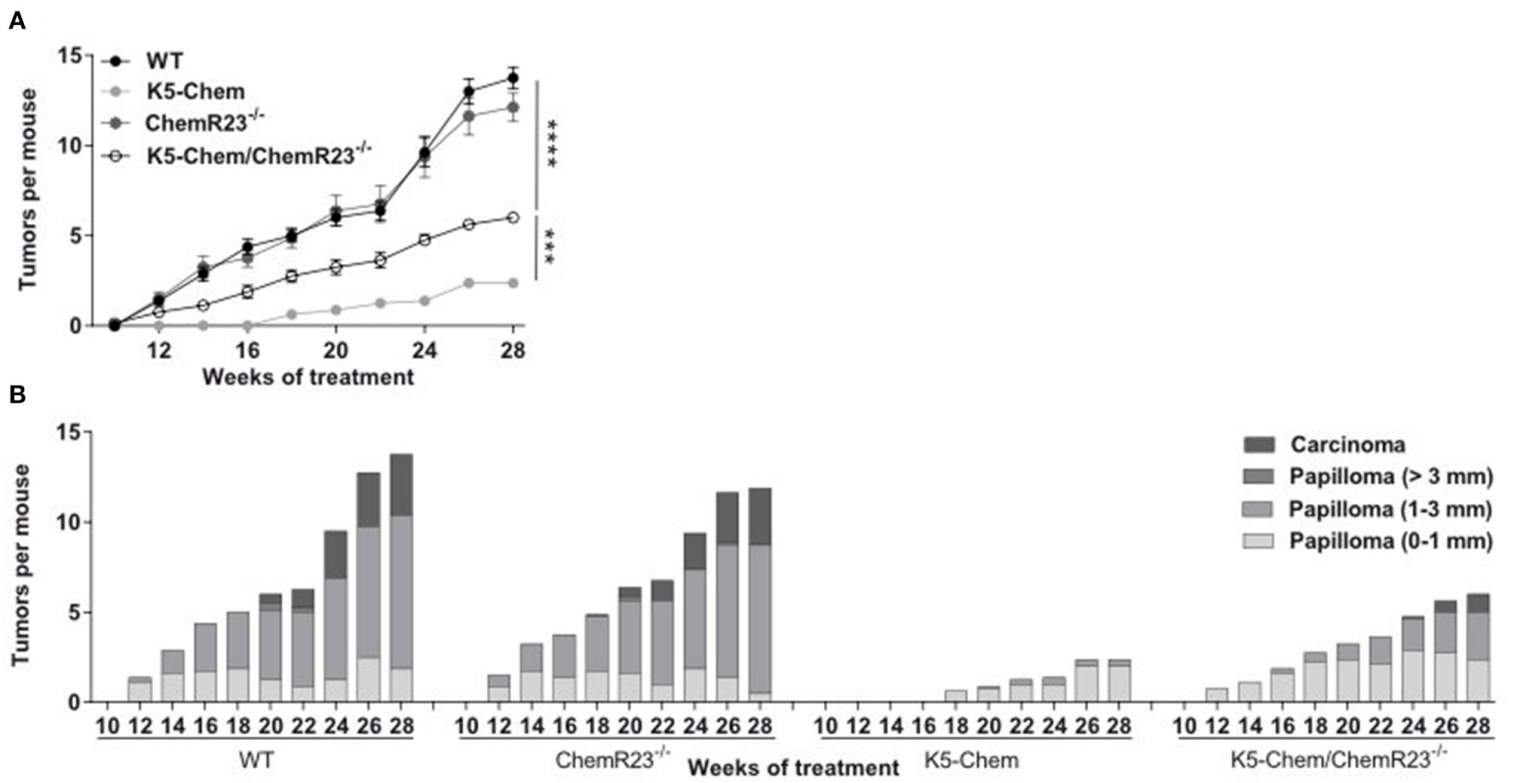
The anti-tumoral properties of chemerin are mediated in part by ChemR23. Control, K5-chemerin, ChemR23^−/−^ and K5-chemerin/ChemR23^−/−^ mice were subjected to the DMBA/TPA chemical carcinogenesis model and the number of tumors per mouse (**A**, mean ± SEM) and size/stage **(B)** were recorded. The data are the compilation of three independent experiments with *n* ≥ 5 mice per group in each experiment. The data are analyzed by two-way ANOVA. ***P < 0.001 and ****P < 0.0001.

In order to determine whether chemerin affects early and/or late stages of the carcinogenesis process in this model, we turned off the expression of chemerin (and EGFP) by doxycycline during part of the procedure. The chemerin levels in plasma, as determined by ELISA, were turned to normal within 2–5 days of the doxycycline treatment (Figure 6), while EGFP fluorescence persisted in the superficial layers of the skin for up to a week (data not shown). The removal of doxycycline from drinking water allowed re-expression of the transgene within 48 h (data not shown). K5-chemerin mice in which chemerin expression was kept on for the first 10 weeks of the DMBA/TPA protocol but was shut down from week 11 behaved exactly as WT mice treated or not with doxycycline (Figure 7A). These data indicate that the early steps of carcinogenesis prior to the development of detectable papillomas are not affected by chemerin. In contrast, when doxycycline was given to mice from 1 week before the initiation of the DMBA/TPA protocol up to the end of week 10, allowing expression of chemerin before the first papillomas became detectable, the number of papillomas developping in K5-chemerin mice was lower than that in wild-type mice, whether or not they had been treated with doxycycline during the first part of the procedure (Figure 7B). The growth of papillomas and their progression to carcinomas were also comparable in the two K5-chemerin groups, while the doxycycline treatment had not effects on the wild-type group. In addition, when the doxycycline treatment was pursued up to the end of week 18, when a significant number of papillomas had appeared in the wild-type groups and the doxycycline-treated K5-chemerin group, reexpression of chemerin prevented further development of new papillomas, and delayed the growth of the ones already present, as well as their progression to carcinomas (Figure 7C). The different timing of tumor development between datasets shown in Figures 4, 7, 8 is attributed to the use of a different batch of DMBA solution for each of these experimental sets and a likely difference in its mutagenic efficacy. Altogether, the data demonstrate that chemerin acts exclusively on the late stages of the carcinogenesis process, including the growth of papillomas once they appeared and their progression to squamous cell carcinomas.

### ChemR23 Mediates Part of the Anti-tumoral Effects of Chemerin

ChemR23 is a fully functional receptor of chemerin while GPR1 displays in our hands moderate signaling abilities in recombinant cell lines (21). We considered therefore ChemR23 as the most likely candidate driving the activities of chemerin in our tumor model. To determine the contribution of ChemR23 in the protective effects of chemerin, we tested the consequences of chemerin overexpression in the DMBA/TPA model, using mice invalidated for ChemR23. K5-chemerin mice invalidated for ChemR23 developed more papillomas than K5-chemerin/ChemR23^+/+^ mice, although not as many as the WT and ChemR23^−/−^ groups (Figure 8). The size of the tumors also remained at an intermediate level. Altogether, we conclude that the effects of chemerin on tumorigenesis are partly mediated by ChemR23, although another mechanism, such as the engagement of the other chemerin receptors, GPR1 or CCRL2, might contribute as well.

## Discussion

Initially described as a chemotactic protein for leukocytes, chemerin is now recognized as a pleiotropic factor, regulating also lipid and glucose metabolism, blood pressure, angiogenesis as well as reproductive functions (15, 45). Chemerin expression was described to be downregulated in many human solid tumors, including squamous cell carcinoma, melanoma and lung and prostate cancer, as well as in mouse cancer models (32, 36, 37). Expression of chemerin by B16 melanoma cells or intratumoral injection of chemerin was reported to reduce tumor growth in syngeneic mice (37). The role of chemerin in various types of cancer and the mechanisms and receptors proposed to mediate its effects have recently been reviewed (32).

ChemR23 is a fully functional receptor of chemerin, leading to inhibition of the cAMP pathway and a strong activation of the phospholipase C/Ca^2+^ and MAP kinase cascades. It is expressed by various leukocyte populations contributing positively or negatively to tumor progression, and is also described on endothelial cells, suggesting a range of potential roles in the frame of tumor development and its control by the host. We observed that ChemR23-deficient mice developed spontaneous skin tumors in areas prone to chronic injury. These tumors were classified as squamous cell carcinoma. Chronic inflammation, and the associated stimulation of tissue repair mechanisms, constitute a critical component of tumor progression, and most frequent cancers arise preferentially from sites of chronic infection, repeated trauma or other causes of persistent inflammatory processes (46, 47). We hypothesized therefore that chemerin could act through its receptor ChemR23 as an agent protecting against the development of skin tumors in a context of chronic inflammation and tissue repair.

Prochemerin is expressed by keratinocytes and downregulated in psoriatic skin (1, 26), suggesting also a role in skin dysfunctions. To better understand the role of chemerin in skin biology and tumorigenesis and test the potential benefit of chemerin analogs to treat or prevent skin cancer, we generated mice overexpressing bioactive chemerin in basal keratinocytes under the control of the keratin K5 promoter. The chemerin form that was selected is the most active form in mouse (and human), ending by -FAFS at the C-terminus (5, 6, 35, 48). It should be noted that two *Rarres2* alleles are found in mice, the products of which differing by the duplication of one glutamine (Q127) in isoform 1 (163 amino acids, NP_001334096) as compared to isoform 2 (162 amino acids, NP_001334097), thereby changing the C-terminal numbering. Expression of the transgene resulted in a significant increase of immunoreactive chemerin both locally in skin but also in the blood flow. Although a significant part of the immunoreactivity in blood corresponds likely to biologically inactive down-products, the chemerin bioactivity remained at values (0.3 nM or higher) in the range of the EC_50_ of the molecule for its main receptors ChemR23 and GPR1. Keratinocyte-released chemerin is therefore expected to display local as well as general effects through these receptors in this transgenic line.

To assess the role of chemerin in a natural context of tumor development, including during the early stages of the process, we used the DMBA/TPA chemical carcinogenesis model. In this model, tumor initiation is achieved by topical administration of DMBA, while promotion is mediated by repeated application of TPA, allowing tumor progression from benign papillomas to malignant carcinomas. Mice overexpressing chemerin in skin displayed delayed development of papillomas, a lower number of papillomas, and a slower progression to large tumors and infiltrating carcinomas. As shown in Figure 5, development of papillomas did not modify expression of EGFP in the epithelium, and the few carcinomas that developed in K5-chemerin mice expressed EGFP as well (data not shown). As the transgene is bicistronic, with EGFP and chemerin expression driven by the same promoter, it is expected that chemerin production was kept as well all along tumor progression. By turning off chemerin production during part of the experimental procedure, we observed that chemerin expression during the early steps of carcinogenesis, namely initiation and promotion, had no effect on the timing of appearance and number of papillomas developing on the treated skin. Importantly, treatment by doxycycline had no consequences on tumor development in the wild-type groups, whatever the timing and length of the treatment. This demonstrates clearly that doxycycline plays a role exclusively through the regulation of chemerin expression in the transgenic groups and has no influence by itself on tumor progression. These results indicate that chemerin has no effect on the immediate responses to DNA damage, such as the DNA repair mechanisms or the elimination of mutant cells by apoptosis, nor on the preferential proliferation of cells bearing driving mutations (such as in *Nras* or *Kras*) promoted by TPA. Rather, chemerin is acting on later events in the carcinogenesis process, leading to the growth of detectable papillomas and their evolution to malignancy.

ChemR23 is the only chemerin receptor displaying full signaling properties and spontaneous skin tumors were observed in ChemR23 KO mice. It was therefore postulated that ChemR23 would likely mediate most of the effects of chemerin on tumorigenesis, and we tested the effect of chemerin in the context of mice invalidated for ChemR23. In our chemical carcinogenesis model, the reversion of the phenotype by ChemR23 invalidation was only partial, suggesting therefore a potential contribution of another chemerin receptor, such as GPR1. Despite its poor signaling properties in recombinant cell lines (21), GPR1 was indeed demonstrated to signal through the RhoA/Rock cascade (49) and to mediate part of the effects of chemerin on the migration and invasion properties of gastric carcinoma cells (50). The contribution of GPR1 should therefore be tested further in the effects of chemerin on the outcome of the DMBA/TPA carcinogenesis protocol. Interestingly, invalidation of ChemR23 has by itself no consequence on the development of papillomas. This is in line with previous results made in a model of B16 melanoma grafts (37). These observations indicate therefore that in two cancer models, the endogenous production of bioactive chemerin is insufficient to contribute an efficient anti-tumoral effect. Nevertheless, these models bypass (B16 grafts) or accelerate considerably (DMBA-TPA model) the slow progression of naturally occurring tumors. The fact that ChemR23-deficient mice develop spontaneous tumors with high frequency shows that the chemerin-ChemR23 system may indeed counteract tumor development in natural situations. In human, in which the longer life span makes the occurrence of various cancer types much more frequent than in mice, such protective effect of the chemerin-ChemR23 system might be more pronounced, as suggested by the reports showing down-regulation of chemerin expression in different cancer types (36, 37).

Tumoral immunity is complex and involves a set of anti-tumoral populations such as cytotoxic T cells, NK cells and M1 macrophages, and pro-tumoral populations including tolerogenic dendritic cells, regulatory T cells, M2 macrophages and myeloid-derived suppressor cells (MDSC), which favor tumor proliferation, vascularization and invasion while inhibiting immune responses (51). Provided the well-established role of the chemerin-ChemR23 system in the recruitment of some of these cell populations, including NK cells, macrophages and dendritic cells (2, 3, 11, 52), these leukocytes might contribute to the observed anti-tumoral properties of chemerin. Previous reports attributed the anti-tumoral effects of chemerin to the recruitment of NK cells (37, 53). In our model, no significant differences in leukocyte populations were observed in the skin of mice in basal conditions (Figures 3D,E), but also at different time points of the DMBA-TPA procedures (not shown), including for the specific leukocyte populations known to be recruited by chemerin, such as NK cells. The lack of recruitment of leukocyte subsets expressing ChemR23 to the skin might be due to the presence of significant amounts of active chemerin in plasma, preventing the formation of a functional gradient between blood and tissues. Such observation was previously made following expression of chemokines in transgenic mice (54). Although we cannot exclude the contribution of the immune system in the anti-tumoral effects of chemerin, as minor leukocyte subpopulations not investigated here might be involved, other mechanisms might also play a role. These alternate mechanisms could include some of the previously described activities of chemerin on different systems and in other cancer models. An anti-tumoral effect of chemerin independently from leukocyte recruitment is supported by a recent report in a model of adrenocortical carcinoma (55), in which chemerin was reported to inhibit cell proliferation by decreasing the activity of WNT/β-catenin and MAPK pathways in tumor cells. Expression of the chemerin receptors ChemR23 and CCRL2 was described in endothelial cells and chemerin was shown to modulate angiogenesis *ex vivo* and *in vivo* (9, 13, 56), which may also influence tumor progression. Chemerin was also described to display antibacterial activities (19, 57), which might result in a modification of the skin microbiome thereby potentially affecting tissue repair mechanisms and tumor development. Although delineating the precise mechanisms supporting the anti-tumoral effects of chemerin will require additional studies, our present results suggest that functional chemerin analogs, such as ChemR23 agonists, might be considered as anti-tumoral agents preventing progression of skin tumors in patients.

## Data Availability Statement

The datasets generated for this study are available on request to the corresponding author.

## Ethics Statement

The animal study was reviewed and approved by Commission d'Ethique du Bien-Etre Animal (CEBEA), Université Libre de Bruxelles, Brussels, Belgium.

## Author Contributions

VW and MP designed the study. ID-V, OD, and VR performed most experiments. JJ, DA, MV, OV, EA-C, and SL contributed to experiments. FL generated the K5-chemerin mouse lines. ID-V wrote the initial draft. MP supervised the study, acquired funding, and wrote the final draft. All authors edited the manuscript.

### Conflict of Interest

SL was employed by company Ogeda S.A. The remaining authors declare that the research was conducted in the absence of any commercial or financial relationships that could be construed as a potential conflict of interest.
