# Corrigendum: Expression of Bioactive Chemerin by Keratinocytes Inhibits Late Stages of Tumor Development in a Chemical Model of Skin Carcinogenesis

**DOI:** 10.3389/fonc.2020.00977

**Published:** 2020-06-23

**Authors:** Ingrid Dubois-Vedrenne, Olivier De Henau, Virginie Robert, Francina Langa, Joaquim Javary, Diana Al Delbany, Olivier Vosters, Edgar Angelats-Canals, Maxime Vernimmen, Souphalone Luangsay, Valérie Wittamer, Marc Parmentier

**Affiliations:** ^1^IRIBHM and Welbio, Université Libre de Bruxelles, Brussels, Belgium; ^2^Centre d'Ingénierie Génétique Murine (CIGM), Institut Pasteur, Paris, France; ^3^Ogeda S.A., Gosselies, Belgium

**Keywords:** chemerin, CMKLR1, ChemR23, chemical carcinogenesis, squamous cell carcinoma

Joaquim Javary was not included as an author in the published article.

The corrected Author Contributions Statement appears below.

## Author Contributions

VW and MP designed the study. ID-V, OD, and VR performed most experiments. JJ, DA, MV, OV, EA-C, and SL contributed to experiments. FL generated the K5-chemerin mouse lines. ID-V wrote the initial draft. MP supervised the study, acquired funding, and wrote the final draft. All authors edited the manuscript.

The authors apologize for this error and state that this does not change the scientific conclusions of the article in any way. The original article has been updated.

